# Regulatory Phenotype, PD-1 and TLR3 Expression in T Cells and Monocytes from HCV Patients Undergoing Antiviral Therapy: A Randomized Clinical Trial

**DOI:** 10.1371/journal.pone.0093620

**Published:** 2014-04-07

**Authors:** Shan-shan Su, Huan He, Ling-bo Kong, Yu-guo Zhang, Su-xian Zhao, Rong-qi Wang, Huan-wei Zheng, Dian-xing Sun, Yue-min Nan, Jun Yu

**Affiliations:** 1 Department of Traditional and Western Medical Hepatology, Third Hospital of Hebei Medical University, Shijiazhuang, China; 2 Department of Infectious Disease, The Fifth Hospital of Shijiazhuang City, Shijiazhuang, China; 3 Department of Liver Disease, Bethune International Peace Hospital, Shijiazhuang, China; 4 Institute of Digestive Disease and Department of Medicine and Therapeutics, State Key Laboratory of Digestive Disease, Li Ka Shing Institute of Health Sciences, The Chinese University of Hong Kong, Hong Kong, China; Glaxo Smith Kline, Denmark

## Abstract

**Background & Aims:**

The cellular immunity has a profound impact on the status of hepatitis C virus (HCV) infection. However, the response of cellular immunity on the virological response in patients with antiviral treatment remains largely unclear. We aimed to clarify the response of peripheral T cells and monocytes in chronic hepatitis C patients with antiviral treatment.

**Methods:**

Patients with chronic hepatitis C were treated either with interferon alpha-2b plus ribavirin (n = 37) or with pegylated interferon alpha-2a plus ribavirin (n = 33) for up to 24 weeks. Frequencies of peripheral regulatory T-cells (Tregs), programmed death-1 (PD-1) expressing CD4^+^ T-cells or CD8^+^ T-cells and toll-like receptor (TLR) 3 expressing CD14^+^ monocytes were evaluated by flow cytometry in patients at baseline, 12 and 24 weeks following treatment and in 20 healthy controls.

**Results:**

Frequencies of Tregs, PD-1 and TLR3 expressing cells were higher in patients than those in control subjects (*P*<0.05). Patients with complete early virological response (cEVR) showed lower Tregs, PD-1 expressing CD4^+^ or CD8^+^ T-cells than those without cEVR at 12 weeks (*P*<0.05). Patients with low TLR3 expressing CD14^+^ monocytes at baseline had a high rate of cEVR (*P*<0.05).

**Conclusions:**

Low peripheral TLR3 expressing CD14^+^ monocytes at baseline could serve as a predictor for cEVR of antiviral therapy in chronic HCV-infected patients. The cEVR rates were significantly increased in the patients with reduced circulating Tregs, PD-1 expressing CD4^+^ or CD8^+^ T-cells.

**Trial Registration:**

Chinese Clinical Trial Registry ChiCTR10001090.

## Introduction

Hepatitis C virus (HCV) infection is a growing public health problem, with roughly 3% of the population infected worldwide [1], [2]. Interferon alpha (IFNα) in combination with ribavirin (RBV) remains a gold standard treatment for chronic HCV infection. However, approximately one-half of chronic hepatitis C genotype 1 patients fail to achieve viral clearance [3]. Thus, it is important to identify the factors that may be valuable in improving antiviral strategy and treatment response.

Both innate and adaptive immunity have a profound impact on status of HCV infection. Impairment of host immunity, especially HCV-specific cellular immune response, may lead to chronic infection [4], [5]. Regulatory T-cells (Tregs) have been shown to be increased in the peripheral blood of patients with chronic HCV infection and can suppress the proliferation of HCV-specific cytotoxic T lymphocytes, which may affect the antiviral treatment response [6]–[8]. Programmed cell death-1 (PD-1), expressing in activated T cells, B cells and monocytes, plays an essential role in regulation of adaptive immune responses [9]. Virus-specific CD8^+^ T cell response generated during acute HCV infection can be gradually exhausted in settings of persistent virus infections by the high expression of PD-1 [10]–[13]. Studies have shown that toll-like receptors (TLRs) can detect the presence of HCV infection through recognition of viral pathogen-associated molecular patterns (PAMPs) and control activation of the adaptive immune responses by inducing dendritic cell maturation [14], [15]. In human monocytes, TLR3 is localized in intracellular endosomal compartments and the agonist of TLR3 represents an attractive target for pursuing the HCV clearance [16]. However, up to now, the effects of Tregs, PD-1 expressing CD4^+^ T-cells or CD8^+^ T-cells and TLR3 expressing CD14^+^ monocytes on virological response in patients treated with IFNα plus RBV remains poorly understood. In this study, we aimed to clarify the role of Tregs, PD-1 and TLR3 in treatment response by assessing the baseline levels and dynamic changes of these immune mediators in patients receiving the combined antiviral treatment.

## Patients and Methods

### Ethics Statement

The protocol for this trial and supporting CONSORT checklist are available as supporting information; see Checklist S1 and Protocol S1. This was a randomized parallel-group study conducted in Shijiazhuang, China. The study protocol conformed to the guidelines of the 1975 Declaration of Helsinki and was approved by the ethics committees of Third Hospital of Hebei Medical University, Shijiazhuang, China, and all patients provided written informed consent. The registration number was ChiCTR-TNRC-10001090.

### Subjects

A total of 70 patients chronically infected with HCV were recruited from Third Hospital of Hebei Medical University, the Fifth Hospital of Shijiazhuang City and Bethune International Peace Hospital (Shijiazhuang, China). The complete date range for patient recruitment and follow-up was from January 2011 to October 2012. HCV infection was diagnosed on the basis of the serum positive antibodies to HCV and the presence of HCV RNA in the plasma. Eligible patients were ≥18 years of age. Subjects meeting with the following criteria were excluded: presence of decompensated cirrhosis, co-infection with human immunodeficiency virus (HIV), hepatitis A, B or D virus, other causes of chronic liver disease or co-morbidities precluding interferon therapy. Twenty age and gender matched healthy subjects were used as controls. Peripheral blood was collected from all of the healthy controls and chronic hepatitis C patients at baseline, 12 and 24 weeks after treatment.

### HCV Antibodies Tests

The serum antibodies to HCV were detected by enzyme linked immunosorbent assay (ELISA) with a commercial detection kit (Livzon diagnostics INC, Zhuhai, China).

### Quantitative Detection of HCV RNA

HCV RNA load was determined using qualitative reverse transcriptase polymerase chain reaction (RT-PCR) assay (Cobas Taqman HCV Test, Roche Diagnostics, Indianapolis, IN) and the low limit quantification was 15 IU/ml.

### Biochemical Assays

Serum alanine aminotransferase (ALT) and aspartate aminotransferase (AST) were detected by an Olympus AU5400 automatic chemical analyzer.

### HCV Genotyping

The HCV genotypes were identified using the HCV genotyping oligochip (Tianjin Third Central Hospital, China) [17].

### Treatments and Assessments of Virological Responses

**Figure 1:**
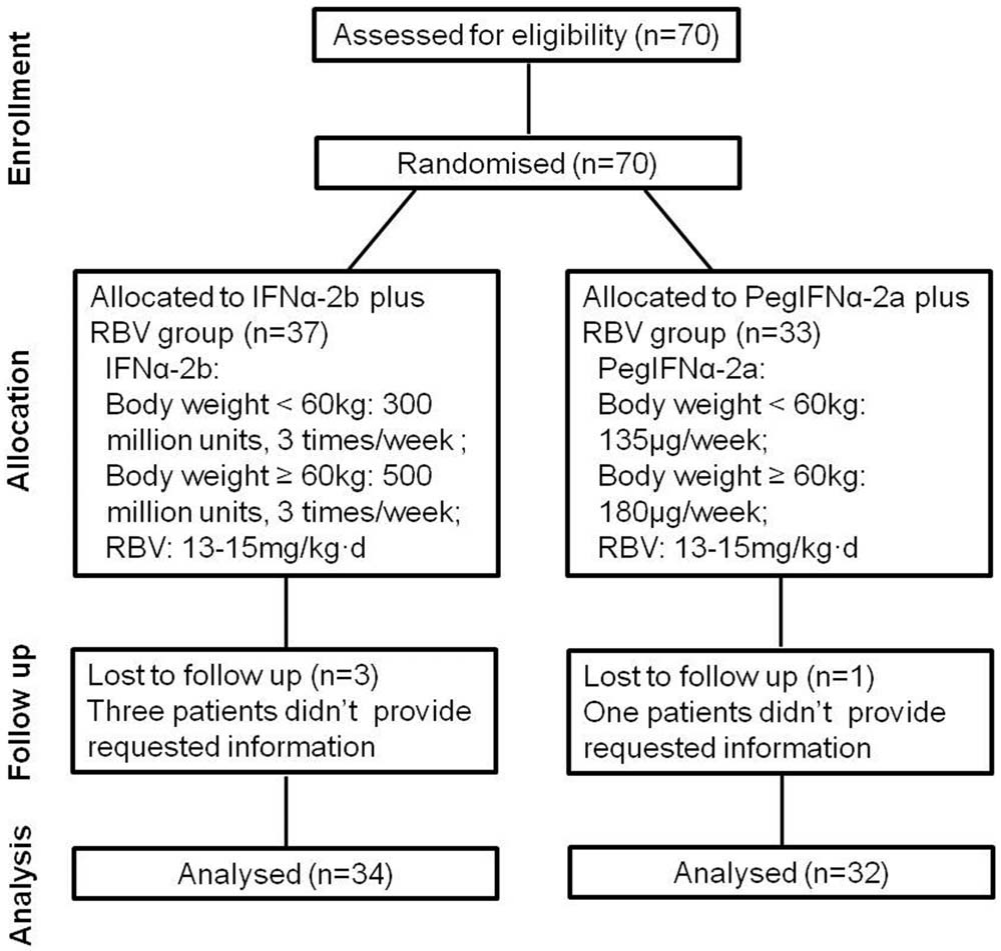
Flow diagram of the progress through the phases of the parallel randomised trial of two groups.

Participants were randomly assigned following simple randomization procedures. Eligible subjects were randomly divided into 2 groups: IFNα-2b plus RBV (IFNα-2b/RBV) group (n = 37), patients underwent therapy of recombinant IFNα-2b (300 million units for body weight<60 kg and 500 million units for body weight≥60 kg, on alternate days) (Beijing Kawin Technology Share-holding Co, Ltd. China) plus weight-based RBV (13–15 mg/kg⋅d) (Zhejiang Chengyi pharmaceuticals Co, Ltd. China); PegIFNα-2a plus RBV (PegIFNα-2a/RBV) group (n = 33), patients received PegIFNα-2a (135 μg/week for body weight<60 kg and 180 μg/week for body weight≥60 kg, subcutaneously) (Pegasys, Roche, Basel, Switzerland) plus weight-based RBV (13–15 mg/kg⋅d) (Fig. 1). Responses to the treatment were assessed by detecting plasma HCV RNA levels at baseline, 12 and 24 weeks after the treatment. Standard definitions of responses were used to evaluate the therapeutic effect [18]. Complete early virological response (cEVR) was defined as undetectable plasma HCV RNA at 12 weeks after treatment. Full details of the trial protocol can be found in the Supplementary Appendix, available with the full text of this article at www.chictr.org/cn/.

### Flow Cytometry Assay for Tregs, PD-1 Expressing T-cells and TLR3 Expressing CD14^+^ Monocytes

Fluorescent dye conjugated antibodies consisted of cell surface monoclonal antibodies CD4-fluorescein isothiocyanate (FITC), CD8-peridinin-chlorophyll-protein complex (PerCP)-Cy5.5, PD-1-phycoerythrin (PE), CD25-PE and CD14-FITC (all from BD Biosciences, San Jose, CA), and intracellular monoclonal antibodies FoxP3-Alexa Flour 647 (BD Biosciences) and TLR3-PE (from eBioscience, San Diego, CA). All appropriate isotype controls were obtained from BD Biosciences. Intracellular staining of FoxP3 was performed according to the manufacturer’s staining kit (BD Biosciences) instructions. Cell surface antigens to identify the PD-1 expression on CD4^+^ or CD8^+^ T-cells were detected with CD4-FITC, CD8-PerCP-Cy5.5 and PD-1-PE. To detect intracellular TLR3 expression in monocytes, cells were stained with surface antibodies CD14-FITC, fixed, permeabilized using FACS™ permeabilizing solution 2 (BD Biosciences) and incubated with TLR3-PE.

At least 100,000 cells were acquired on FACS CantoII (Becton Dickinson, San Jose, CA) and analyzed using Cellquest software. For data analyses, an initial lymphocyte gate was set based on side scatter (SSC)/forward scatter (FSC) and additional gates introduced as required. Results were present as the percentage of positively stained cells within the gated population.

### Statistics Analysis

The statistical power calculation was performed based on predictive significance of cEVR to sustained virological response (SVR) achievement. The target sample size gave 95% power at the 5% significance level to detect a difference of 58% in SVR rates (82.2% *vs* 24.2%) between the cEVR and non-cEVE patients according to the outcome of our completed clinical trial. Categorical data were expressed as numbers or proportions of subjects with the specific features. The chi-square test was used to compare categorical data. Continuous variables not normally distributed were summarized as medians and ranges, and Nonparametric Mann-Whitney *U* test was used to compare the differences between two groups. Kruskal-Wallis *H* test was used for comparison of differences between three groups and further comparisons between any two groups within these multiple groups were conducted using Nemenyi method. A mixed-effects model analysis of variance was applied to evaluate the time and group effects as well as the time by group interaction. If the overall *P*-value was significant, we used a step-down test based on the Bonferroni correction method to determine pairwise differences. Correlations between the variables were calculated using Spearman rank order correlations. Multivariate logistic regression analysis was performed to identify independent predictors of cEVR. All *P*-values were two-tailed, and were considered significant when lower than 0.05. Data were analyzed using SPSS 16.0 software package (v. 16.0; SPSS Inc., Chicago, IL).

## Results

### Patient Characteristics

At baseline, there were no significant differences between IFNα-2b/RBV and PegIFNα-2a/RBV treatment groups for the demographic and clinical characteristics, including gender, age, body mass index, serum ALT, AST, HCV RNA levels, percentages of different contaminated ways and HCV genotypes (Table 1). The age and gender between chronic hepatitis C patients and healthy controls were also no significant difference (data not shown).

**Table 1:** Baseline Characteristics of the Subjects.

Parameter	IFNα-2b/RBV (n = 37)	PegIFNα/RBV (n = 33)	*P*-value
Gender (M/F)	16/21	16/17	0.660
Age, mean±SD	47.7±12.1	48.5±13.7	0.767
Body mass index (kg/m^2^), mean±SD	23.3±2.9	23.4±2.7	0.938
ALT (IU/L), median (range)	68.5 (15–259)	66.6 (10–191)	0.457
AST (IU/L), median (range)	56.4 (17–189)	53.6 (15–228)	0.452
HCV RNA (IU/ml), median (range)	5.4×10^5^ (403–6.0×10^8^)	7.6×10^5^ (1220–2.8×10^7^)	0.723
Possible route of contamination			
Transfusion, n (%)	23 (62.2)	24 (72.7)	0.348
Previous surgery, n (%)	4 (10.8)	3 (9.1)	0.811
Stomatologic treatments, n (%)	1 (2.7)	1 (3.0)	0.935
Others or unknown, n (%)	9 (24.3)	5 (15.2)	0.338
HCV genotypes (1b/2a)	30/5	31/1	0.110

Abbreviations: IFN: Interferon; RBV: Ribavirin; ALT: Alanine transaminase; AST: Aspartate transaminase.

Among 70 patients, 61 (87.1%, 61/70) of them were infected with HCV genotype 1b and 6 (8.6%, 6/70) patients were infected with HCV genotype 2a. The HCV genotype data were missing in 3 (4.3%, 3/70) patients.

### Treatment Responses

As 4 cases who didn’t provide requested information at 12 weeks after treatment were missing, 66 subjects were included in the efficacy analysis for cEVR assessment (34 patients treated with IFNα-2b/RBV and 32 cases with PegIFNα-2a/RBV). Among 34 patients treated with IFNα-2b/RBV, 25 (73.5%, 25/34) achieved cEVR, and 25 (78.1%, 25/32) underwent PegIFNα-2a/RBV treatment achieved cEVR. There was no significant difference in the cEVR incidences between the two groups (*P* = 0.663).

We evaluated the potential relationship between HCV genotypes and early response patterns (cEVR or non-cEVR). Among 66 subjects included in the efficacy analysis for cEVR assessment (60 patients infected with HCV genotype 1b and 6 cases with HCV genotype 2a), 45 (75.0%, 45/60) patients with HCV genotype 1b infection achieved cEVR, while 5 (83.3%, 5/6) patients infected with HCV genotype 2a achieved cEVR.

### Tregs and Virological Response

**Figure 2:**
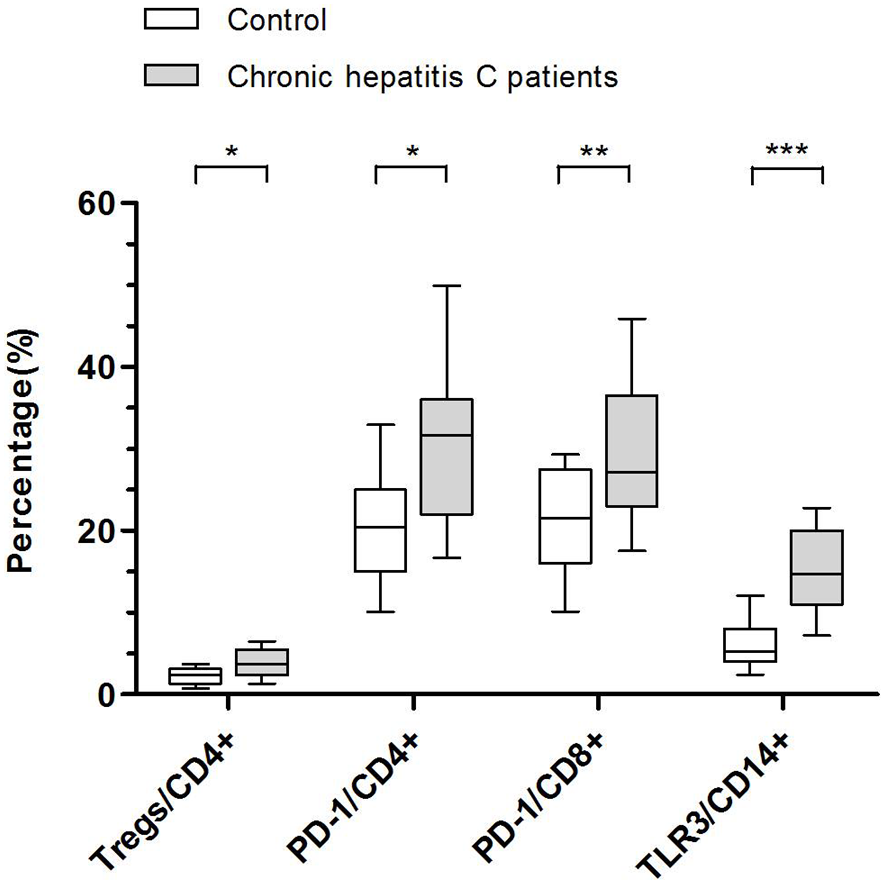
Proportions of Tregs, PD-1 expressing CD4^+^ T-cells or CD8^+^ T-cells and TLR3 expressing CD14^+^ monocytes. Boxes represent the interquartile range and horizontal lines inside each box represent the median. The vertical lines from the ends of each box encompass the extreme data points. Proportions of Tregs, PD-1 expressing CD4^+^ T-cells or CD8^+^ T-cells and TLR3 expressing CD14^+^ monocytes were measured by flow cytometry and data were presented by percentage. *P*-values were determined by Mann-Whitney test. *P<0.05, **P<0.01, ***P<0.001.

**Figure 3:**
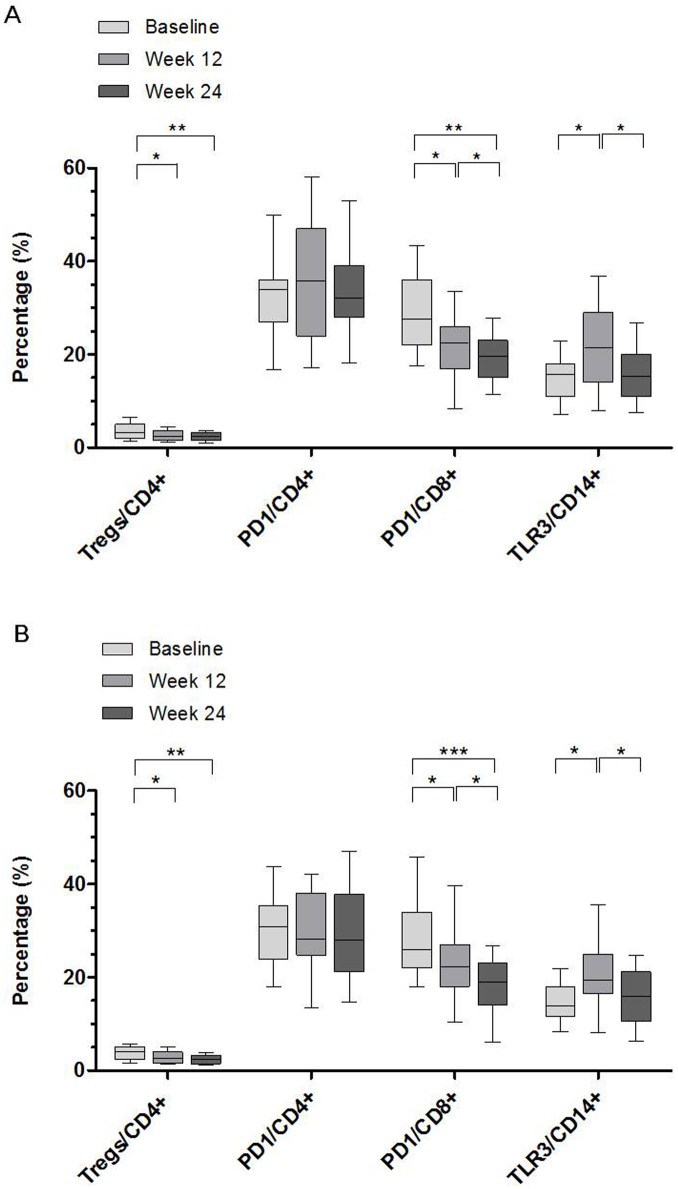
Proportions of Tregs, PD-1 expressing CD4^+^ T-cells or CD8^+^ T-cells and TLR3 expressing CD14^+^ monocytes in patients treated with (A) IFNα-2b/RBV or (B) PegIFNα-2a/RBV. Boxes represent the interquartile range and horizontal lines inside each box represent the median. The vertical lines from the ends of each box encompass the extreme data points. Proportions of Tregs, PD-1 expressing CD4^+^ T-cells or CD8^+^ T-cells and TLR3 expressing CD14^+^ monocytes were measured by flow cytometry and data were presented by percentage. *P*-values were determined by Kruskal-Wallis *H* test and Nemenyi test. *P<0.05, **P<0.01, ***P<0.001.

**Figure 4:**
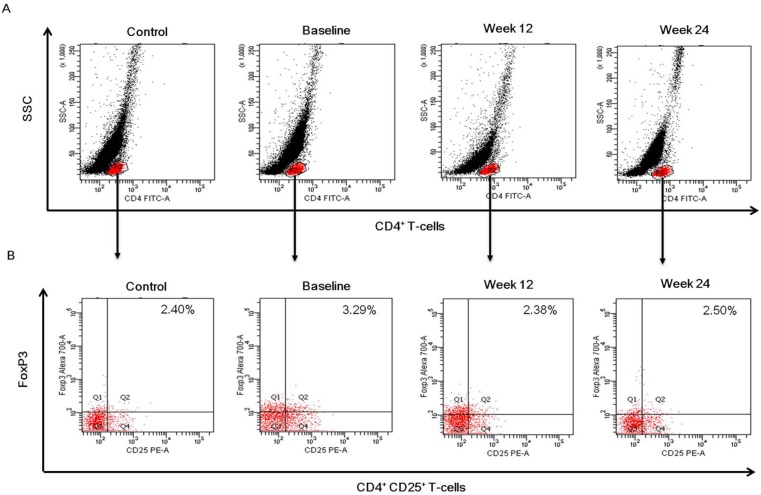
Representative dot plots of Tregs frequencies in peripheral CD4^+^ T-cells in healthy controls and chronic HCV-infected patients. (A) CD4^+^ lymphocytes were gated by positive staining of CD4 and side scatter (SSC). (B) Dot plots illustrating CD4^+^CD25^+^FoxP3^+^ Tregs in CD4^+^ cells. Cells were stained with monoclonal antibodies to CD4, CD25 and FoxP3. The lymphocyte subpopulation was selected on the basis of forward scatter (FSC) and SSC from peripheral blood cells. The percentage of positive cells is indicated in the upper right region.

The effect of different types of interferon combined with RBV on peripheral CD4^+^CD25^+^FoxP3^+^ Tregs proportion in CD4^+^ T-cells was assessed. The Tregs frequency was higher in chronic hepatitis C patients than that in the controls (*P* = 0.031) (Fig. 2), which was significantly decreased at 12 weeks after treatment with IFNα-2b/RBV (*P* = 0.015) (Fig. 3A) or PegIFNα-2a/RBV (*P* = 0.034) (Fig. 3B). No further change was observed at 24 weeks compared with 12 weeks following treatment. With respect to the Tregs levels, no significant difference was observed between IFNα-2b/RBV and PegIFNα-2a/RBV groups in any time point (*P*>0.05). A representative flow cytometry dot plot of Tregs proportion in CD4^+^ T-cells was shown in Fig. 4A and 4B.

**Figure 5:**
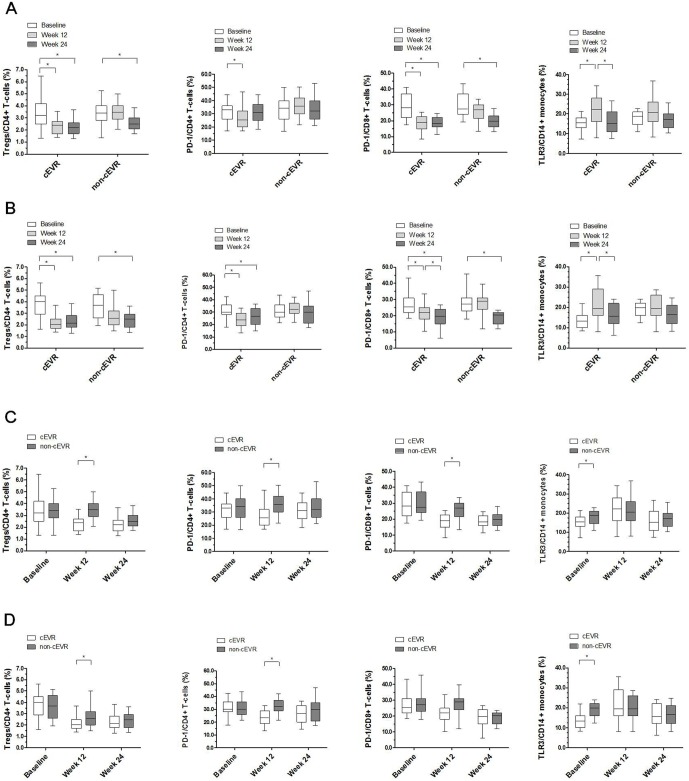
Proportions of Tregs, PD-1 expressing CD4^+^ T-cells or CD8^+^ T-cells and TLR3 expressing CD14^+^ monocytes at three time points treated with (A) IFNα-2b/RBV or (B) PegIFNα-2a/RBV and in cEVR or non-cEVR patients treated with (C) IFNα-2b/RBV or (D) PegIFNα-2a/RBV. Boxes represent the interquartile range and horizontal lines inside each box represent the median. The vertical lines from the ends of each box encompass the extreme data points. Proportions of Tregs, PD-1 expressing CD4^+^ T-cells or CD8^+^ T-cells and TLR3 expressing CD14^+^ monocytes were measured by flow cytometry and data were presented by percentage. *P*-values were determined by mixed model and Bonferroni correction method. *P<0.0167.

As compared with the baselines, the Tregs frequency was significantly decreased at 12, 24 weeks in cEVR patients (*P*<0.001) and at 24 weeks in non-cEVR cases (*P* = 0.001 and *P* = 0.015, respectively) after treatment with IFNα-2b/RBV or PegIFNα-2a/RBV (Fig. 5A and 5B). Futhermore, Tregs frequency was significantly lower in cEVR patients than that in non-cEVR cases after 12 weeks of medication (*P*<0.001 and *P* = 0.015, respectively) (Fig. 5C and 5D), whereas no significant difference in the proportion was found at baseline and 24 weeks after therapy (Fig. 5C and 5D).

### PD-1 Expressing CD4^+^ Or CD8^+^ T-cells and Virological Response

**Figure 6:**
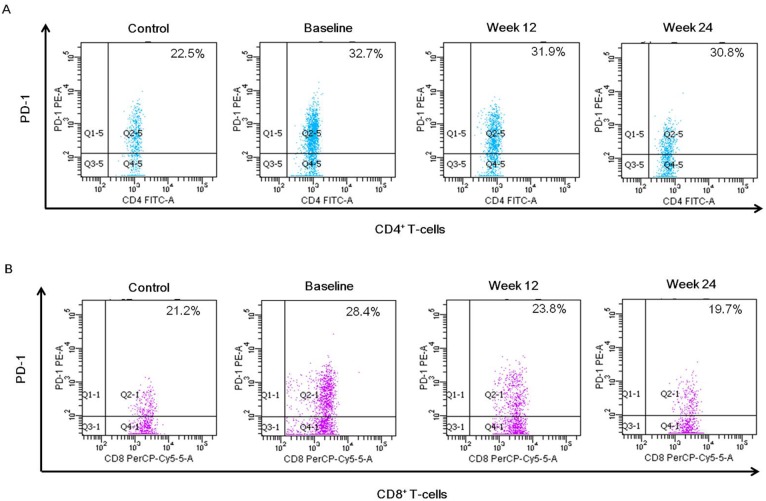
Representative dot plots of PD-1 expressing CD4^+^ T-cells or CD8^+^ T-cells in healthy controls and chronic HCV-infected patients. (A) Dot plots illustrating PD-1 expressing CD4^+^ cells and (B) CD8^+^ cells. Cells were stained with monoclonal antibodies to CD4, CD8 and PD-1. The lymphocyte subpopulation was selected on the basis of forward scatter (FSC) and side scatter (SSC) from peripheral blood cells. CD4^+^ or CD8^+^ lymphocytes were gated by positive staining of CD4 or CD8 and SSC. The percentage of positive cells is indicated in the upper right region.

PD-1 expression on T-cell subsets was determined by the percentage of PD-1 positive cells. The PD-1 expressing CD4^+^ T-cells (*P* = 0.012) or CD8^+^ T-cells (*P* = 0.004) were significantly higher in chronic hepatitis C patients than those in healthy controls at baseline (Fig. 2). Compared with its baseline level, PD-1 expressing CD8^+^ T-cells was decreased after 12 weeks of therapy in patients received IFNα-2b/RBV (*P* = 0.035) or PegIFNα-2a/RBV (*P* = 0.015), and further reduced at 24 weeks after treatment compared with its 12 weeks level (*P* = 0.034 and *P* = 0.029, respectively) (Fig. 3A and 3B). However, the PD-1 expressing CD4^+^ T-cells did not show significant change after antiviral treatment. In addition, there was no significant difference of the PD-1 expressing CD4^+^ T-cells or CD8^+^ T-cells between IFNα-2b/RBV and PegIFNα-2a/RBV groups in any parallel time point (*P*>0.05). A representative flow cytometry dot plot of PD-1 expressing CD4^+^ or CD8^+^ T-cells was shown in Fig. 6A and 6B.

By analyzing the relationship between PD-1 level and virological response, compared with the baseline levels, PD-1 expressing CD4^+^ T-cells were markedly decreased after 12 weeks of therapy with IFNα-2b/RBV (*P* = 0.004) or PegIFNα-2a/RBV (*P*<0.001) in cEVR patients, but it did not show significant change in non-cEVR cases (Fig. 5A and 5B). As respect to PD-1 expressing CD8^+^ T-cells, we found that it’s frequency was significantly decreased at 12, 24 weeks in cEVR patients (*P*<0.001) and at 24 weeks in non-cEVR cases (*P* = 0.009 and *P* = 0.006, respectively) after treatment with IFNα-2b/RBV or PegIFNα-2a/RBV (Fig. 5A and 5B). Compared with non-cEVR patients, frequencies of PD-1 expressing CD4^+^ (*P* = 0.005 and *P* = 0.002, respectively) in two treatment groups and PD-1 expressing CD8^+^ T-cells (*P* = 0.001) in IFNα-2b/RBV treated group were significantly lower in patients who achieved cEVR after 12 weeks of therapy (Fig. 5C and 5D), though no differences were seen at baseline and 24 weeks after treatment (Fig. 5C and 5D).

### TLR3 Expressing CD14^+^ Monocytes and Virological Response

**Figure 7:**
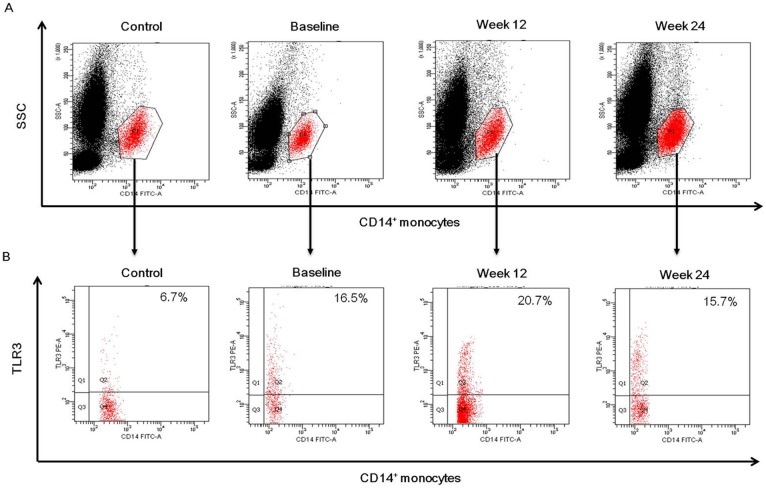
Representative dot plots of TLR3 expressing CD14^+^ monocytes in healthy controls and chronic HCV-infected patients. (A) CD14^+^ monocytes were gated by positive staining of CD14 and side scatter (SSC). (B) Dot plots illustrating TLR3 expressing CD14^+^ cells. Cells were stained with monoclonal antibodies to CD14 and TLR3. The percentage of positive cells is indicated in the upper right region.

TLR3 expressing CD14^+^ monocytes, determined by the percentage of TLR3 positive cells, were higher in chronic hepatitis C patients than those in healthy controls (*P*<0.001) (Fig. 2), and further increased after 12 weeks of treatment (*P* = 0.033 and *P* = 0.018, respectively), but returned to baseline level at 24 weeks in the two treatment groups (Fig. 3A and 3B). No significant difference was presented in TLR3 expressing CD14^+^ monocytes between IFNα-2b/RBV and PegIFNα-2a/RBV groups in any parallel time point (*P*>0.05). A representative flow cytometry dot plot of TLR3 expressing CD14^+^ monocytes was shown in Fig. 7A and 7B.

TLR3 expressing CD14^+^ monocytes were significantly increased after 12 weeks of treatment (*P*<0.001), and restored close to the baseline levels at 24 weeks in cEVR patients of the two treatment groups, but this changes were not significant in non-cEVR cases (Fig. 5A and 5B). Notably, the baseline TLR3 expressing CD14^+^ monocytes were significantly lower in patients achieved cEVR than those with non-cEVR (*P* = 0.009 and *P* = 0.011, respectively) (Fig. 5C and 5D), whereas there was no relationship between TLR3 expressing CD14^+^ monocytes and virological response at 12 and 24 weeks after treatment in the two groups (Fig. 5C and 5D).

### Predict Factors of Treatment Response

Variables that may be related to treatment response were assessed by multivariate logistic regression analysis, including baseline laboratory parameters, consisting of Tregs, PD-1expressing CD4^+^ T-cells or CD8^+^ T-cells, TLR3 expressing CD14^+^ monocytes, serum viral load, ALT and AST, as well as the use of interferon types. It was found that low proportion of baseline TLR3 expressing CD14^+^ monocytes was a single predictor of cEVR in chronic hepatitis C patients with antiviral treatment [odds ratio (OR)  = 2.11, 95% confidence intervals (CI) (1.15–3.88), *P* = 0.023].

### Lack of Association of Tregs, PD-1 and TLR3 with HCV Genotype, Viral Load, ALT and AST

Relationships of the baseline levels and the dynamic changes during treatment of Tregs, PD-1 expressing CD4^+^ T-cells or CD8^+^ T-cells and TLR3 expressing CD14^+^ monocytes with HCV genotypes were analyzed. No difference was found between HCV genotype 1b and 2a infected patients at each time point (data not shown). Moreover, there was no correlation of Tregs, PD-1 expressing CD4^+^ T-cells or CD8^+^ T-cells and TLR3 expressing CD14^+^ monocytes with baseline HCV load, ALT and AST levels as well (data not shown).

## Discussion

Patients who achieved cEVR have the best chance of achieving SVR [19], [20]. In our study, the cEVR rates were 73.5% and 78.1% in patients treated with IFNα-2b/RBV and PegIFNα-2a/RBV, respectively. The results were better than others reported on the early pivotal clinical trials, in which only 50%–66% patients infected with HCV achieved cEVR [21]–[23]. The reasons for this disparity might arise from host IL-28B genetic variation (rs12979860 CC genotype) [24] and low body weight in Chinese population which were associated with interferon sensitivity and treatment-induced viral clearance.

A favorable treatment response includes a strong cellular immune response against HCV antigens [25], [26]. To seek the role of cellular immune response in chronic hepatitis C patients with antiviral treatment, we investigated the dynamic changes of peripheral Tregs, PD-1 expressing T-cells and TLR3 expressing CD14^+^ monocytes in the patients. In the present study, proportions of Tregs and PD-1 expressing CD4^+^ T-cells or CD8^+^ T-cells were significantly increased in the patients at baseline as compared with the controls, indicating that the impaired cellular immune function might contribute to chronic HCV infection. It was reported that HCV infection had a high propensity to develop persistent viremia and was associated with ineffective viral-specific CD4^+^ and CD8^+^ T-cells responses [27], [28]. The cause of T-cells failure might relate to up-regulation of CD4^+^CD25^+^FoxP3^+^ Tregs [6] and PD-1 expressions [11], [13], [29]–[32] which inhibited HCV-specific CD4^+^ and CD8^+^ T-cells function.

Feld et al. [33] has suggested that the immune-modulatory activity of interferon and ribavirin therapy may be associated with enhancing T effector cells response to HCV, thus, the impaired cellular immunity by persistent HCV infection might be partially restored by interferon and ribavirin administration. In our study, there was a significant decrease of Tregs and PD-1 expressing CD8^+^ T-cells in chronic hepatitis C patients at 12 weeks after treatment compared with their baseline levels, indicating that IFN/RBV treatment could restore HCV-specific T-cells response by reducing circulating CD4^+^CD25^+^FoxP3^+^ Tregs and PD-1 expressing CD8^+^ T-cells. Furthermore, the decrease proportions of Tregs and PD-1 expressing CD4^+^ T-cells or CD8^+^ T-cells were more obvious in cEVR patients than those in non-cEVR cases. According to Thimme et al. [34], [35], T cell exhaustion contributed to the failure of about half of HCV-specific CD8^+^ T cell responses and the antiviral efficacy of HCV-specific CD8^+^ T cells was linked to surface expression of PD-1. Other studies had also reported that PD-1 was up-regulated on total CD4^+^ and CD8^+^ T cells in patients with chronic infection compared with normal subjects, and antiviral therapy that led to sustained elimination of circulating HCV RNA was associated with down-regulation of PD-1 on a wide range of cells [36], [37]. Besides PD-1 modulation, activation of Tregs also involved in impairment of HCV-specific T effector cells. Langhans’s study proposed that antiviral therapy is likely to shift the functional balance in the immune system between Tregs and T effector cells towards more efficacious effector responses in chronic hepatitis C patients. Our finding was in accordance with Langhans report [38], which indicated that interferon combined with ribavirin therapy could enhance proliferation of T effector cells as well as inhibit functions of Tregs, thus, reverses Treg-mediated suppression of T effector cells in chronic hepatitis C.

Treatment with IFNα-2b/RBV or PegIFNα-2a/RBV for 12 weeks, the frequencies of circulating Tregs and PD-1 expressing CD4^+^ in two groups or PD-1 expressing CD8^+^ T-cells in IFNα-2b/RBV group in patients who achieved cEVR were significantly lower than those in patients without cEVR. For PegIFNα-2a/RBV treated group, the frequencies of PD-1 expressing CD8^+^ was also lower in cEVR patients, but the *P* value was higher than 0.0167, perhaps on account of small sample size. Therefore, we speculated that Tregs and PD-1 could inhibit the responder cells population and function, which correlated with the risk of developing non-response. In contrast, patients who showed relatively abrogated capacity of Tregs and PD-1 might achieve cEVR and recover from HCV infection. This demonstrated that the suppression of Tregs and PD-1 might correlate with cEVR as it could generate an effective immune response and which provide a possible therapeutic target for immune modulation. However, whether the decrease in the frequency of Tregs and PD-1 expressing CD4^+^ or CD8^+^ T-cells were the necessary causes of cEVR warranted further investigation. No significant differences of the Tregs and PD-1 expressing CD4^+^ T-cells or CD8^+^ T-cells were found between IFNα-2b/RBV and PegIFNα-2a/RBV treated groups at any parallel time either from cEVR or non-cEVR, indicating that the immunomodulatory effects of IFNα-2b and PegIFNα-2a were comparable.

Activation of TLRs can induce both innate and adaptive immunity [39]. TLR3 signaling is induced by double-stranded (ds) RNA, a molecular signature of viruses, and is mediated by the TIR domain-containing adaptor-inducing IFN-β (TRIF) adaptor molecule. Thus, TLR3 plays an important role in the host response to viral infections [40]. The innate immune response activated through recognition of viral PAMPs by TLR3 leads to IFN-β induction, which in turn increases the interferon stimulated genes (ISGs) expression. Thus far, TLR3 expression in peripheral blood has not been well analyzed in chronic hepatitis C patients under antiviral treatment. Monocytes in peripheral blood were identified as the cells responding to TLRs stimulation [41]. In present study, we found that the level of TLR3 expressing CD14^+^ monocytes was elevated in chronic hepatitis C patients and further increased at 12 weeks, but returned to baseline level at 24 weeks after treatment with IFNα-2b/RBV or PegIFNα-2a/RBV. This change was more significant in cEVR patients than that in non-cEVR ones. This suggested that the anti-HCV treatment could increase TLR3 expressing CD14^+^ monocytes to enhance antiviral activity at early stage of treatment and restore the cell number and function to baseline level as HCV was cleared. An important finding coming from our TLR3 analysis was that lower peripheral TLR3 expressing CD14^+^ monocytes at baseline was an independent predictor for cEVR. In keeping with our findings, Hashad et al. [22] and Yuki et al. [23] found that pretreatment up-regulation of hepatic TLR3 expression was a predictive factor for non-respond. HCV infection could activate monocytes via TLRs pathway and elevate the expression of TLR3 as our study found, but pre-existing monocytes activation was a factor mediating reduced responses to TLR3 stimulation [42]. Thus, infection of monocytes by HCV may account for a defective response to HCV infection. Furthermore, the endogenous IFN system activation in the liver before treatment not only was ineffective in HCV clearance but also hampered further response to exogenous IFN plus RBV [37]. These reasons might explain why the level of TLR3 expressing CD14^+^ monocytes was lower in cEVR patients than that in non-cEVR ones, but further and expanded investigations were needed to clarify the underlying mechanisms. Therefore, the baseline level of peripheral TLR3 expressing CD14^+^ monocytes might serve as a predictor of response to antiviral therapy and have a substantial impact on treatment decision-making.

## Conclusions

In summary, the present study suggested that low peripheral TLR3 expressing CD14^+^ monocytes at baseline was a novel predictor for early virological response of antiviral therapy in chronic HCV-infected patients. The patients treated with interferon plus ribavirin could achieve completely early virological response through the modulation of peripheral CD4^+^CD25^+^FoxP3^+^ regulatory T-cells, PD-1 expressing CD4^+^ or CD8^+^ T-cells and TLR3 expressing CD14^+^ monocytes.

## Supporting Information

Checklist S1 **CONSORT 2010 checklist information to report the randomized parallel-group study.** The study process, reported sections and topics, checklist item numbers, content and described sections in this manuscript were described.
(DOC)

Protocol S1 **Study protocol in English.** Study design and exclusion criteria for this study was presented in English in this protocol.
(DOC)

Protocol S2 **Study protocol in Chinese.** Study design and exclusion criteria for this study was presented in Chinese in this protocol.
(DOC)
