# Supplementary material for: Regulatory Phenotype, PD-1 and TLR3 Expression in T Cells and Monocytes from HCV Patients Undergoing Antiviral Therapy: A Randomized Clinical Trial
Source: PLoS One. 2014 Apr 7;9(4):e93620. doi: 10.1371/journal.pone.0093620 (PMC3977904; doi:10.1371/journal.pone.0093620)
Supplement: Protocol S1 — Study protocol in English. Study design and exclusion criteria for this study was presented in English in this protocol. (DOC) [file pone.0093620.s002.doc]

**Study Protocol**

**Study design**: Randomized parallel control

**Inclusion criteria**: 1. Baseline examinations are consistent with the diagnosis of CHC; 2. Compensated liver diseases.

**Exclusion criteria**: 1. History or other evidence of decompensated liver disease (Coagulopathy, hyperbilirubinemia, hepatic encephalopathy, hypo-albuminemia, ascites, and bleeding from esophageal varices are conditons consistant with decompensated liver disease; patients with cirrhosis in Child-Pugh score > 6); 2. Co-infection with active hepatitis B, hepatitis D and/or human immuno-deficiency virus (HIV); 3. Neutrophil count < 1000 cells/mm^3^, platelet count < 50000 cells/mm^3^ at screening or Hemoglobin < 8 g/dL at screening; 4. Sign or symptoms of hepatocellular carcinoma, patients with a value of alpha-fetoprotein > 100 ng/mL; 5. Pregnant or breast-feeding females (positive pregnancy test immediately prior to initiation of therapy), male partners of women who are to be pregnant; 6. Serum creatinine level > 1.5 ULN at screening; 7. History of drug allergy on IFN or any compound of IFN; 8. History of drug and alcohol abuse; 9. History of autoimmune disease; History of severe psychiatric disease, especially depression; History of thyroid disease poorly controlled on prescribed medications; History of severe retinal disease; History of malignant tumor; History of chronic pulmonary disease with insufficient function; History of severe cardiac or digestive disease; History of disease aggravating anaemia; 10. Other issues that investigators consider unsuitable for this trial.

**Participant age**: ≥ 18 years

**Gender**: Both

**Study execute time**: From 1-1-2011 to 31-12-2012

**Subjects:**

PegIFNα-2a/RBV group:

Sample size: 35

Intervention: The subjects will be treated as follows: Pegasys 135μg/wk (for body weight < 60kg) or 180μg/wk (for body weight ≥ 60kg) plus Ribavirin 13-15mg/kg/day for 44 weeks after HCV RNA undetectable/clearance.

IFNα-2b/RBV group:

Sample size: 35

Intervention: The rest patients will be treated as follows: IFNα-2b 300MU/2 days (for body weight < 60kg) or 500MU/2 days (for body weight ≥ 60kg) plus Ribavirin 13-15mg/kg/day for 44 weeks after HCV RNA undetectable /clearance.

Health control group:

Sample size: 20

Intervention: None

**Date of approved by Ethics Committee of 3rd Hospital of HeiBei Medical University:** Oct 13th, 2010.

**Randomization Procedure**: Participants were randomly assigned following simple randomization procedures.

**Methods**: All patients were evaluated at baseline, 12, 24 weeks following treatment. On each visit time, routine hematological workup, biochemical assays, qualitative HCV RNA and frequencies of peripheral regulatory T-cells (Tregs), programmed death-1 (PD-1) expressing CD4^+^ T-cells or CD8^+^ T-cells and toll-like receptor (TLR) 3 expressing CD14^+^ monocytes were tested.

**Virological Response:** Complete early virological response (cEVR) was defined as undetectable plasma HCV RNA at 12 weeks after treatment.

**Statistics analysis:**

The chi-square test was used to compare categorical data. Continuous variables not normally distributed were summarized as medians and ranges, and nonparametric Mann-Whitney *U* test was used to compare the differences between the groups. Correlations between the variables were calculated using Spearman rank order correlations. Multivariate logistic regression analysis was performed to identify independent predictors of cEVR. All *P*-values were two-tailed, and were considered significant when lower than 0.05. Data were analyzed using SPSS 16.0 software package (v. 16.0; SPSS Inc., Chicago, IL).

**Countries of recruitment and research settings****:**

Department of Traditional and Western Medical Hepatology, Third Hospital of Hebei Medical University, Shijiazhuang, China

Department of Infectious Disease, the Fifth Hospital of Shijiazhuang City, Shijiazhuang, China

Department of Liver Disease, Bethune International Peace Hospital, Shijiazhuang, China

Department of Liver Disease, First hospital of Hebei medical University, Shijiazhuang, China

Department of Liver Disease, Handan Infectious Diseases Hospital, Handan, China

**Data collection Institution：**Third Hospital of Hebei Medical University

**Data management Institution：**Third Hospital of Hebei Medical University

**Data analysis Institution**：Third Hospital of Hebei Medical University
