# Supplementary material for: Regulatory Phenotype, PD-1 and TLR3 Expression in T Cells and Monocytes from HCV Patients Undergoing Antiviral Therapy: A Randomized Clinical Trial
Source: PLoS One. 2014 Apr 7;9(4):e93620. doi: 10.1371/journal.pone.0093620 (PMC3977904; doi:10.1371/journal.pone.0093620)
Supplement: Protocol S2 — Study protocol in Chinese. Study design and exclusion criteria for this study was presented in Chinese in this protocol. (DOC) [file pone.0093620.s003.doc]

**慢性丙型病毒性肝炎优化治疗方案的临床研究I （初治患者）**

承 担 单 位 ：**河北医科大学第三医院**

协 作 单 位 ：白求恩国际和平医院

河北医科大学第一医院

石家庄市第五医院

邯郸市传染病医院

项目负责人：**南 月 敏**

项 目 编 号 ：10276102D

项目起止年月：2010.01-2012.12

**一、研究目的**

通过前瞻性、开放性、随机临床研究，建立慢性丙型肝炎初治患者的优化治疗方案，通过优化治疗提高丙型肝炎的SVR率，改善慢性丙型肝炎患者的预后及生活质量。

**1．患者评估及方案选择**

①患者评估：感染时间、基因型、病毒载量、胰岛素抵抗情况、合并疾病情况等；

②方案选择：

A 样本量：130

干预措施：PegIFNα-2a联合RBV，PegIFNα-2a 180μg（体重≥60kg）皮下注射，1次/周，RBV 13-15mg/kg/day。若不耐受PegIFNα-2a 180μg或体重<60kg患者可调整为135μg。

B 样本量：130

干预措施：普通干扰素联合RBV，IFNα-2b 500MU（体重≥60kg）皮下注射，隔日1次，RBV 13-15mg/kg/day。若不耐受IFNα-2b 500MU或体重<60kg患者可调整为 300MU。

**二、研究方案实施：**

根据2004年中华医学会肝病学分会、中华医学会传染病与寄生虫病学分会制定的《丙型肝炎防治指南》诊断标准，选择慢性丙型肝炎/代偿期肝硬化患者260例，根据基线情况随机入组。

**1.病例纳入标准：**

(1)年龄：16～65岁；

(2)诊断明确的慢性丙型肝炎；

(3)代偿期肝硬化（Child-Pugh分级A级）；

(4)依从性好，定期复查血常规、肝肾功能、甲状腺功能、HCV-RNA定量等；

(5)由受试者或其家属（监护人）签署同意参加本研究的书面知情同意书。

**2.病例排除标准：**

1. 失代偿性肝病史（如：失代偿性肝病伴凝血紊乱，高胆红素血症，肝性脑病，低蛋白血症，腹水和食管静脉曲张出血，肝硬化病人Child-Pugh分数>6）；
2. 合并HBV、HDV、HIV感染；
3. 中性粒细胞<1.0×10^9^/L，血小板<50×109/L，血红蛋白<80g/L；
4. 确诊为肝癌或疑有肝癌或AFP>100ng/ml者；
5. 妊娠及哺乳期妇女；
6. 筛选时肌酐值>1.5倍正常参考值上限（ULN）；
7. 对干扰素或该制剂的任何成分有过敏史；
8. 有吸毒或酗酒史；
9. 自身免疫性疾病、严重精神性疾病、甲状腺疾病、重度视网膜疾病、恶性肿瘤史、严重肺疾病、消化系统疾病、心脏疾病、任何使贫血风险增加的疾病或病史；
10. 其它由研究者判断不适合进入本研究者。

**3.疗效评估**

参照2004年中国《丙型肝炎防治指南》、2007年APASL《丙型肝炎病毒感染诊断与治疗共识》及2009年AASLD新版《丙型肝炎诊断，管理和治疗》。

**3.1治疗前评估：**

(1)病史及体格检查：感染途径、病程、体重指数（kg/m^2^）、肝脾大小；

(2)血液学检查：血常规、肝肾功能、血糖、血沉、风湿三项、肝病自身抗体、甲状腺功能、AFP、抗-HCV、HCV RNA、HCV基因型；

(3)细胞免疫功能检测：用流式细胞术检测对照组（20例）及CHC患者（70例）不同治疗组在治疗前、治疗第12、24周时外周血CD4+CD25+FoxP3+ Treg细胞在CD4+ T细胞、PD-1表达阳性CD4+和CD8+ T细胞、TLR3表达阳性CD14+单核细胞所占的百分率。

(4)细胞凋亡、炎症及纤维化因子检测：TNF-α、Fas、IL-6和TGF-β，ELISA法统一检测。

(5)超声检查：肝脏大小、形态，脂肪肝程度，占位性病变，门脉宽度及血流；脾脏大小。

(6)其他：心电图、胸片及眼底检查。

**3.2治疗中监测：**

(1)第1个月内每周检测1次血常规，以后若血常规正常者，可每3个月检查1次；白细胞降低者，给予升白药物治疗同时，每2周或4周检查1次；

(2)每4-8周检测血清生化学指标；

(3)第4周、12周、24周、48周、56周、68周定量检测血清HCV-RNA；治疗结束时行抗HCV检测；

(4)每3个月检测甲状腺功能、自身抗体、细胞免疫功能、炎症及纤维化相关因子；

(5)每6个月检测甲胎蛋白、腹部超声、胸片、眼底检查。

**3.3治疗结束后随访：**

随访12周、24周检测血常规、血生化、HCV-RNA、甲状腺功能、自身抗体、细胞免疫功能、炎症及纤维化相关因子；24周行甲胎蛋白及腹部超声检查。

**4.治疗应答情况评价：**

**(1) 抗病毒治疗应答**

- 快速病毒学应答（rapid virological response，RVR）：4周时血清HCV RNA定量检测小于最低检测限。
- 早期病毒学应答（early virological response，EVR）：12周时血清HCV RNA定性检测阴性（或定量检测小于最低检测限），或定量检测降低2个对数级（Log10）以上。完全早期病毒学应答（complete early virological response，cEVR）指治疗12周时血清HCV RNA定性检测阴性（或定量检测小于最低检测限）。部分早期应答（partial early virological response，pEVR）治疗12周时血清HCV RNA，定量检测降低2个对数级（Log10）)。
- 治疗结束时病毒学应答（end of treatment virological response，ETVR）：治疗结束时HCV RNA定量检测小于最低检测限。
- 持续病毒学应答（sustained virological response，SVR）：即治疗结束至少随访24周时，定性检测HCV RNA阴性(或定量检测小于最低检测限)。
- 无应答（non-response，NR）：指从未获得EVR、ETVR及SVR者。
- 复发（relapse）：指治疗结束时定性检测HCV RNA为阴性(或定量检测小于最低检测限)，但停药后HCV RNA又变为阳性。
- 病毒学反弹（breakthrough）：治疗期间曾有HCV RNA载量降低或阴转，但尚未停药即出现HCV RNA载量上升或阳转。

(2)多因素回归分析：基线因素、白细胞减少、血红蛋白和肌酐水平、细胞免疫功能及相关细胞因子等与RVR、EVR、ETVR及SVR的关系。

(3)停药原则：

- 干扰素停药原则

中性粒细胞绝对计数≤500 cells/mm^3^或血小板< 30000 cells/mm^3^；研究者认为需要停药的其它情况。

- 利巴韦林停药原则

治疗期间出现Hb≤8g/dl时；治疗期间干扰素停药；研究者认为需要停药的其它情况。

(4)不良反应的处理：治疗期间出现流感样症状、骨髓抑制、肝功能异常、甲状腺功能异常等情况时给予对症处理**。**

**5.治疗疗程：**依据患者的应答情况，均于HCV RNA转阴后再治疗44周，随访24周。
